# The Evolution of an Osmotically Inducible *dps* in the Genus *Streptomyces*

**DOI:** 10.1371/journal.pone.0060772

**Published:** 2013-04-01

**Authors:** Paul D. Facey, Matthew D. Hitchings, Jason S. Williams, David O. F. Skibinski, Paul J. Dyson, Ricardo Del Sol

**Affiliations:** Institute of Life Science, College of Medicine, Swansea University, Swansea, United Kingdom; University of Utah, United States of America

## Abstract

Dps proteins are found almost ubiquitously in bacterial genomes and there is now an appreciation of their multifaceted roles in various stress responses. Previous studies have shown that this family of proteins assemble into dodecamers and their quaternary structure is entirely critical to their function. Moreover, the numbers of *dps* genes per bacterial genome is variable; even amongst closely related species - however, for many genera this enigma is yet to be satisfactorily explained. We reconstruct the most probable evolutionary history of Dps in *Streptomyces* genomes. Typically, these bacteria encode for more than one Dps protein. We offer the explanation that variation in the number of *dps* per genome among closely related *Streptomyces* can be explained by gene duplication or lateral acquisition, and the former preceded a subsequent shift in expression patterns for one of the resultant paralogs. We show that the genome of *S. coelicolor* encodes for three Dps proteins including a tailless Dps. Our *in vivo* observations show that the tailless protein, unlike the other two Dps in *S. coelicolor,* does not readily oligomerise. Phylogenetic and bioinformatic analyses combined with expression studies indicate that in several *Streptomyces* species at least one Dps is significantly over-expressed during osmotic shock, but the identity of the ortholog varies. *In silico* analysis of *dps* promoter regions coupled with gene expression studies of duplicated *dps* genes shows that paralogous gene pairs are expressed differentially and this correlates with the presence of a *sigB* promoter. Lastly, we identify a rare novel clade of Dps and show that a representative of these proteins in *S. coelicolor* possesses a dodecameric quaternary structure of high stability.

## Background

Almost two decades after the first Dps protein (PexB) was characterized in *E. coli* [1], [2], there has been a continued effort to identify and characterize homologous genes in other prokaryote genomes. Subsequently, Dps proteins have been found in almost all the bacterial groups, including archaea [3]. As a result, the literature now contains an abundance of data on Dps proteins; providing a wealth of knowledge pertaining to their structure [4], interaction with DNA [5] and their importance in the stress response [6]. Interestingly, there is an apparent evolutionary link between the iron sequestering Ferritin (and bacterioferritin) proteins and Dps proteins [7] and, moreover there also appears to be ferritin-like proteins that share functional properties of Dps [8]. Indeed, a few studies have elucidated the crystalline structure of a few Dps proteins [9], [10] and have shown that like ferritins, Dps proteins assemble into oligomers (albeit dodecamers as opposed to 24mers) and their overall three-dimensional shape is entirely critical to their function [11]. Moreover, it is because of the smaller nature of Dps oligomers (when compared to ferritins) that Dps proteins are often referred to as mini-ferritins; as opposed to maxi-ferritins [12]. However, despite similarities between these two protein families, they can be distinguished based on examination of their secondary structure. For example, Dps have a small central helix (often called the BC helix) which is absent in ferritins. Similarly, ferritins possess a helix towards their C-terminus that is absent in Dps [13]. However, both ferritins and Dps both form similar tertiary structures.

The current literature suggests that Dps proteins provide macromolecule protection either by oxidizing and storing ferrous iron in a bioavailable form or by binding and physically shielding DNA. Iron detoxification, which Dps proteins contribute to through the abatement of the Fenton reaction, occurs at the ferroxidation centre. These sites lie at the interface between two anti-parallel subunits ([10] and references therein) and are therefore found within the hollow, inner cavity of the self-assembled dodecamer. However, not all Dps proteins have ferroxidase activity and not all bind DNA [14]. To the exterior of the dodecamer, the variable length N- and C-terminal tails of each monomer have been implicated in DNA binding and dodecamer assembly [15], [16]. For example, removal of the N and C terminal tails of *Mycobacterium smegmatis* Dps-1 prevents assembly of the dodecamer. However, other Dps proteins are “tailless” and still assemble into dodecamers [17] suggesting that, although in some genera the tails are important for dodecamer assembly, this is not always the case. However, whilst differences in tail length may reflect important variation in structure (and possibly function), this has only been studied in a few bacteria; studies on the evolution and origin of these tails appears scant. Indeed, until recently, the focus of many Dps studies has been on their role in protection during nutrient limitation and under oxidative conditions, usually through the abatement of Fenton Chemistry [18]. However, there now appears some recognition of the multifaceted roles of *dps* genes (see [19], [20], [21], [22], [23]) and that different Dps proteins within the same genome may be capable of different functions. With increasing numbers of completely sequenced microbial genomes, multiple *dps* homologs within the same species are now being identified and their structures and functions elucidated [24], [25], [26]. Having more than one *dps* gene per genome is common amongst bacteria [27]. However, for many genera, variation in the number of *dps* per genome amongst closely related species is yet to be satisfactorily explained. This is exemplified in species of mycobacteria. Gupta *et al.,* [28] and Roy *et al.,* [29] characterized two *dps* genes in free-living *Mycobacterium smegmatis*. Yet, remarkably, *dps* homologs are absent in the genomes of closely related, but pathogenic, *M. leprae*, *M. bovis* and *M. tuberculosis*. Additionally, more intricate studies are now characterizing the control of *dps* at the transcriptional level [27], [30] and, not only is there evidence for multiple sigma factor complexes contributing to the transcription of *dps* [30], [31] but, it also appears that expression of these genes may be driven by suites of different sigma factors e.g. *msdps1* [31] and *msdps2* [27]. Together, this suggests that the evolutionary history of *dps* in many genera contains duplications, losses and possible lateral acquisitions.

The aim of the present study is to investigate the evolutionary history of the *dps* genes in *Streptomyces*. Recent studies have identified three *dps* genes (herein named *dpsA_Sc_*, *dpsB_Sc_* and *dpsC_Sc_*) in the genome of the model actinomycete *S. coelicolor* [19]. *dpsA_Sc_* has been shown to be part of the well characterized, osmotically-induced Sigma B regulon and is significantly overexpressed as a result of osmotic stress and heat shock [19], [30]. Similarly, *dpsC_Sc_* is also mildly induced during heat shock. However, despite contributing to nucleoid compaction, *dpsB_Sc_* appears not to be induced during osmotic nor heat shock. Phylogenetic analysis indicates that horizontal gene transfer (HGT) and gene duplication are plausible explanations for the distribution of *dps* genes among *Streptomyces.*

## Results

### Distribution of Dps_Sc_ orthologs in Bacteria

**Figure 1:**
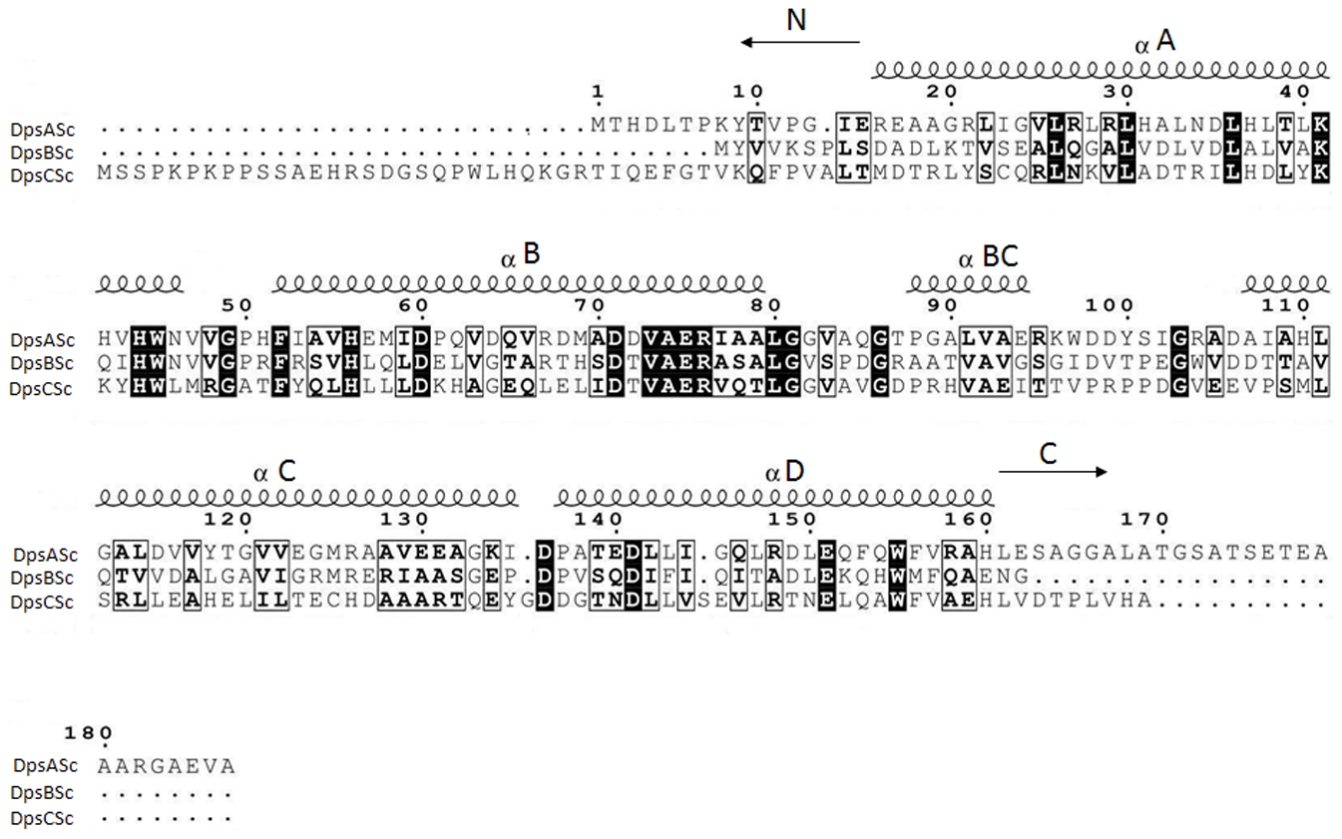
Amino acid alignment of *S. coelicolor* Dps proteins. Amino acid alignment of the three S. *coelicolor* Dps proteins showing the position of the five characteristic Dps helices along with the position of the different length N- and C-terminal tails.

We used the three *Streptomyces coelicolor* Dps proteins as BLASTP queries for homology searches among Bacterial and Archaeal lineages. Our searches yielded 1120 unique protein sequences and included sequences from 299 completely sequenced prokaryote genomes across 8 different phyla. Retrieved sequences varied in annotation and included Dps family ferritin, starvation induced *dps*, ferritin, DNA-binding Dps and hypothetical proteins. All sequences were confirmed to be Dps by the possession of signature amino acids and the helical pattern characteristic of Dps protein’s secondary structure [6], [19] (figure 1 provides a schematic representation showing the positions of the helices in the three Dps proteins in *S. coelicolor*). The results of our homology searches showed that the number of *dps* genes per genome was variable – even amongst closely related species. However, most (75%) genomes encoded for only one Dps protein, with 18% encoding for two Dps proteins, 5% encoding for three Dps proteins and less than one percent of all genomes scrutinized had more than 3 *dps* genes.

Interestingly, there was no significant (P>0.05; Mann-Whitney) distributional bias in the number of homologs per genome among the 8 phyla. Similarly in *Streptomyces,* the number and distribution of *dps* genes among closely related species was unequal. Moreover, the genomes of *S. coelicolor* and *S. ghanaensis* are within a minority of bacteria that contain 3 *dps* genes.

### Variation in tail length of bacterial Dps and assembly and stability of *S. coelicolor* Dps

We uncovered significant differences in the distributions of tail lengths in Dps proteins within and among bacterial genera. Interestingly, all tail lengths appeared to occur randomly throughout the bacterial phyla - and this variation is also evident in *Streptomyces.* Moreover, not only does the *S. coelicolor* genome encode for three Dps proteins, each of these has different tail lengths (Figure 1). However, as expected from the variable distribution of Dps in *Streptomyces*, this pattern is not conserved. Indeed, in our dataset, the presence of three *dps* genes in the same genome, each encoding for a protein with a different secondary structure is unique. In other genomes that have three *dps* (e.g. *S. ghanaensis*) at least two Dps homologs have similar tail lengths. For example, in *S. ghanaensis*, two Dps are orthologous to DpsB_Sc_ and have both short N- and C-terminal tails and the other is orthologous to DpsA_Sc_ and has a longer N-terminal tail (compared to C-terminal tail). The most frequent tail length arrangement (83% of all Dps proteins in Streptomycetes) is short/negligible tails, making this secondary structure almost ubiquitous in *Streptomyces.* Only 30% of Dps proteins in *Streptomyces* have longer C-terminal tails and 20% with longer N-terminal tails.

**Figure 2:**
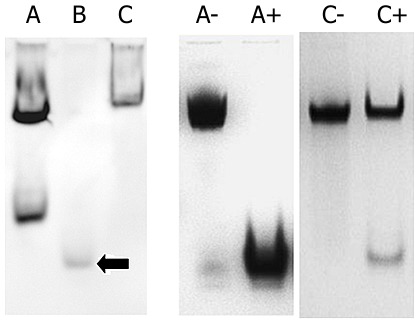
A: Immunoblot of a native PAGE gel. Immunoblot showing the *in vivo* oligomeric state of DpsA_Sc_, DpsB_Sc_ and DpsC_Sc_ overexpressed using a thiostrepton inducible promoter. Overexpression of DpsA (lane A), DpsB (lane B) and DpsC (lane C) from a thiostrepton inducible promoter. Black arrow indicates the non-dodecameric DpsB. **B:** **Coomassie blue stained native PAGE gel assessing the stability of assembled Dps_Sc_ oligomers.** Coomassie blue stained native PAGE gel showing the stability of assembled *S. coelicolor* Dps oligomers after incubation with 8 M urea. Lane A−  =  DpsA_Sc_ without 8 M urea, lane A+  =  DpsA_Sc_ + 8 M urea, Lane C−  =  DpsC_Sc_ without 8 M Urea, lane C+  =  DpsC_Sc_ + 8 M urea.

A native protein Western blot (Figure 2A) revealed that DpsA_Sc_ and DpsC_Sc_ readily assemble into higher oligomeric states *in vivo* in contrast to DpsB_Sc_ that does not. Moreover, our *in vivo* observations of DpsA_Sc_ shows that it appears to be present in two major species on a 7% native PAGE gel (lanes 1–3). The mobility of the lower DpsA oligomer indicates that it is significantly smaller than a dodecamer (but larger than DpsB) whilst the upper species is believed to be a dodecamer.

*S. coelicolor* Dps oligomers also display differences in their resistance to denaturation. Of the two Dps that assemble into dodecamers (DpsA and DpsC), we found that DpsC dodecamers are significantly more resistant to denaturation than those of DpsA. The native PAGE gels (Figure 2B) indicate that whilst the dodecamer of DpsA is denatured by 8 M urea, the DpsC oligomer is much more stable. Even after extended incubation with 8 M urea, the DpsC dodecamer exhibits very little disassembly - indicating that the molecular interactions maintaining the oligomeric structure are very strong.

### The distribution of orthologous protein clusters and tail lengths

**Figure 3:**
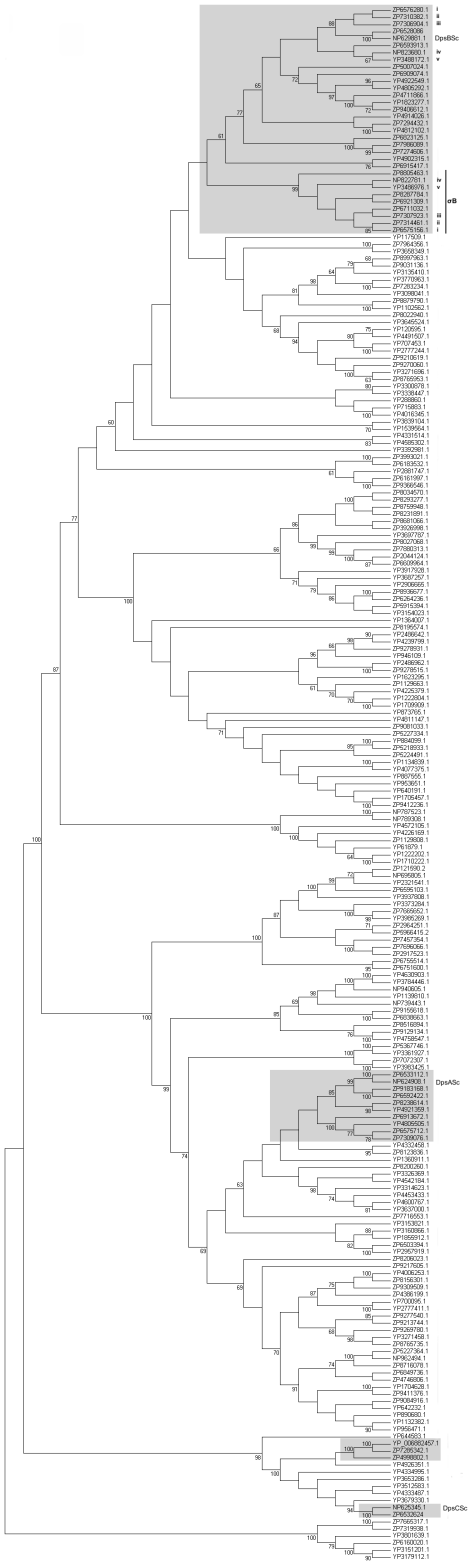
Majority rule consensus phylogenetic tree of Actinobacterial Dps orthologous proteins. Maximum-likelihood reconstructed phylogenetic tree of Actinobacterial Dps proteins. Indicated are three orthologous protein clusters (shaded boxes). DpsA_Sc_, DpsB_Sc_ and DpsC_Sc_ indicates the position of *S. coelicolor* Dps in the tree. Paralogous gene pairs of DpsB in *Streptomyces* are indicated using matched Roman numerals. σB indicates those proteins where a putative *sigB-like* promoter was identified. Bootstrap values >60% are indicated next to major nodes.

Highlighted on the Maximum-likelihood reconstructed phylogeny of Actinobacterial Dps sequences (figure 3) are three distinct clades. These correspond to the three *S. coelicolor* orthologous Dps protein clusters. Remarkably, proteins that are orthologous to DpsC_Sc_ are rare in bacteria, and even rarer in *Streptomyces* – certainly, orthologs of DpsC_Sc_ were found in only five Streptomycetes. Thus, this very narrowly distributed clade contains very interesting Dps proteins. Similarly, orthologs of DpsA_Sc_ are rare in *Streptomyces*, and, this is suggestive of non-lineal inheritance. In contrast, many Streptomycetes possess an ortholog of DpsB_Sc_ (indicated in figure 3 by the large cluster of *Streptomyces*) and the origin of this large cluster of orthologous proteins has a very deep node - indicative, maybe, of very early divergence in the *Streptomyces*. In all cases we found that the GC content of *dps* genes was consistent with the local GC content of neighbouring genes and also the genome average.

### Duplication and expression of Dps in *Streptomyces* genomes

**Figure 4:**
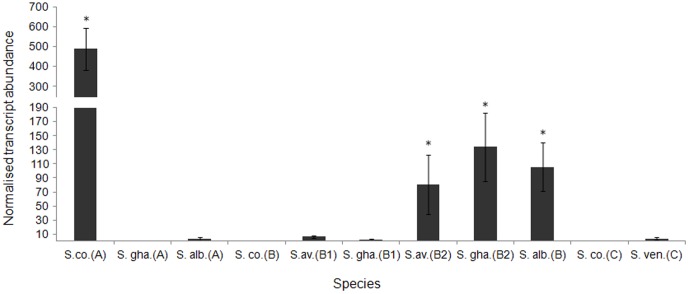
Normalized fold change in *dps* transcript abundance among five *Streptomyces* in response to osmotic stress. q RT PCR monitoring of *dps* transcript abundance after 1 hour incubation with 250 mM KCl in S. co.  =  *S. coelicolor*; S. gha  =  *S. ghanaensis*; S. alb  =  *S. albus*; S. av  =  *S. avermitilis*; S. ven.  =  *S. venezuelae*. Relationships to *S. coelicolor dps* orthologs are indicated in parentheses. *dps* transcript abundance is normalized to principal sigma factor *hrdB*. Paralogous gene pairs are sequentially numbered. Presence of a *sigB-like* promoter motif upstream of the ORF is indicated with an asterisk. Error bars represent standard deviation of the mean normalized transcript abundance. A broken Y-axis has been used.

Our expression analysis shows that there is a trend for at least one *dp*s per genome to be upregulated during osmotic stress although the identity of the upregulated ortholog differs among *Streptomyces* (Figure 4). Moreover, possession of an osmotically regulated *dps* appears to have arisen in many genomes after gene duplication. The ML phylogenetic tree identifies a duplication of *dps* genes in a few *Streptomyces* genomes. The genomes of *S. avermitilis*, *S. scabies*, *S. ghanensis, S. griseoflavus, S. viridochromogenes* and *S. sviceus* contain two highly similar copies (pairwise mean percentage similarity  =  97% ± 1.5 S.D) of a *dps* that is orthologous to *dpsB_Sc._* Interestingly, expression analysis after osmotic upshock shows that only one member of each paralogous gene pair is induced (Figure 4) and these group together within the tree (Figure 3).

**Figure 5:**
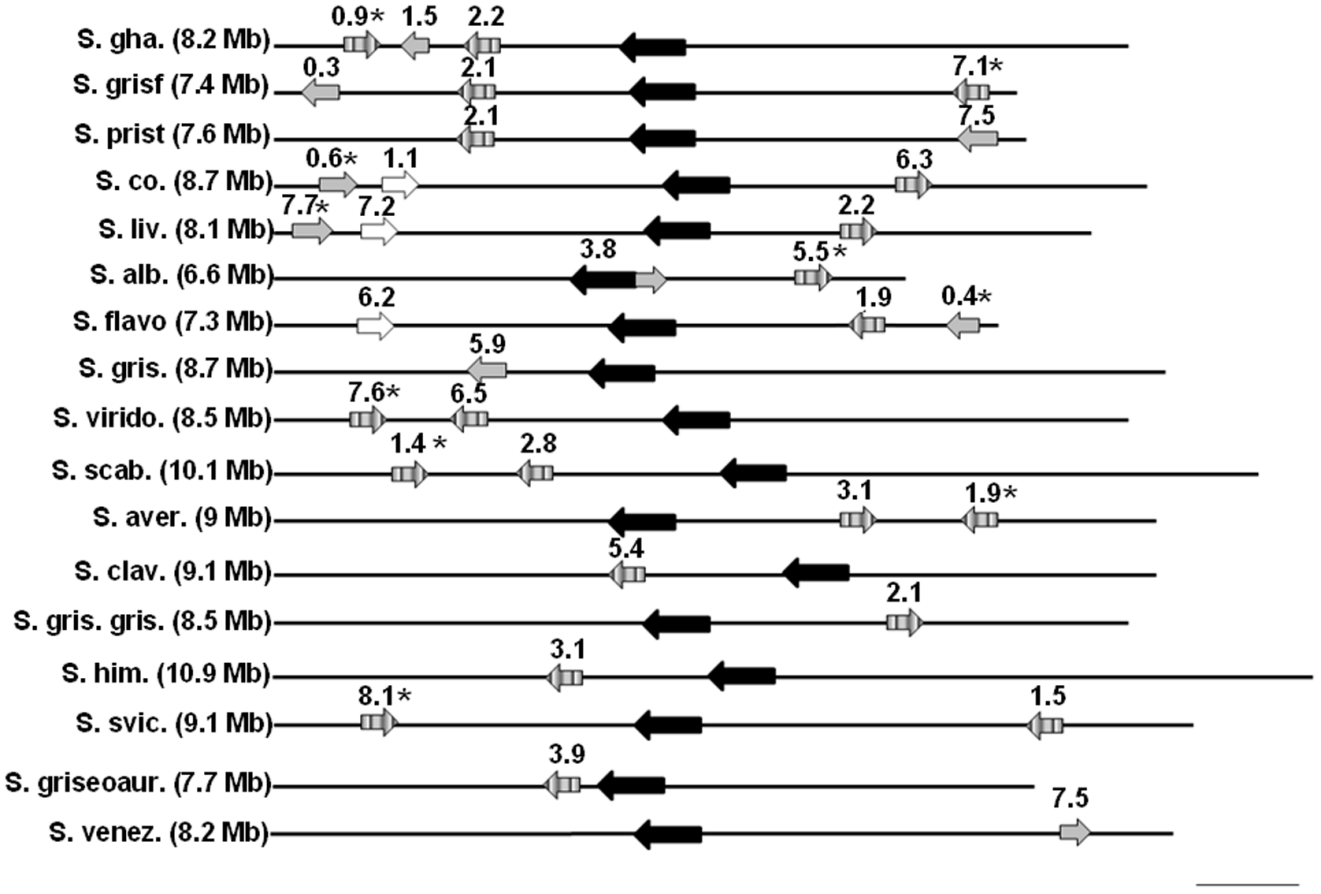
*dps* location maps among 17 Streptomycete chromosomes. Approximate chromosome location of *dps* orthologs in 17 Streptomycete chromosomes. Species names are abbreviated to the left of the diagram: S. gha  =  *S. ghanaensis*; S. griseof  =  *S. griseoflavus*; S. prist  =  *S. pristinaespiralis*; S. co.  =  *S. coelicolor*; S. liv.  =  *S. lividans*; S. alb  =  *S. albus*; S. flavo  =  *S. flavogriseus*; S.gris  =  *S. griseus*; S. virido  =  *S.viridochromogenes*; S. scab  =  *S. scabies*; S. aver  =  *S. avermitilis*; S. clav  =  *S. clavuligerus*; S. gris. gris  =  *S. griseus* subsp. *griseus*; S. him  =  *S. himastatinicus*; S. svic  =  *S. sviceus*; S. griseoaur  =  *S. griseoaurantiacus*; S. venez  =  *S. venezuelae*. Chromosomes were orientated based on the centrally located initiator protein (black arrow). Light grey arrows represent orthologs of *dpsA_Sc_*, clear arrows represent orthologs of *dpsC_S_c*, striped arrows represent orthologs of *dpsB_Sc_*. Figures above arrows represent approximate chromosome location in Mb. Figures in parentheses  =  chromosome size. Asterisks indicate those genes where we identified *sigB-like* promoter motifs upstream of ORFs. Scale bar  =  1 Mb.

Figure 5 summarizes the results of our searches for *sigB-like* promoter motifs upstream of *dps* genes in 17 completely sequenced Streptomycetes. Although *dpsA_Sc_* is transcribed from a *sigB*-like promoter, our approach of mapping putative *sigB* promoters using an *in silico* approach revealed that not all orthologs of *dpsA_Sc_* in other species possess a recognizable SigB-dependent promoter. Furthermore, for species that lack an ortholog of *dpsA_Sc_,* or where this ortholog is present but lacks a *sigB*-like consensus promoter sequence upstream, the genome often contains an alternative ortholog with a putative SigB-dependent promoter. In species where we identified a duplicated *dps,* in all cases one of these copies had a *sigB* promoter motif. Moreover, in four species tested, we confirmed that *dps* genes with a *sigB*-like promoter motif upstream of their ORF are significantly upregulated during osmotic stress (Figure 2).

### Gene synteny and chromosomal location of *dps* in *Streptomyces*

In order to provide insights into an evolutionary history of *dps*, which may have included gene duplications and lateral acquisitions in *Streptomyces*, we performed synteny comparisons among all completely sequenced and assembled Streptomycete genomes. In addition, we also compared the chromosome locations of *dps* genes in 17 streptomycete genomes. Our comparisons of gene location (Figure 5) showed that orthologs of *dpsA_Sc_* and *dpsC_Sc_* are generally located outside of the core genome (i.e. in the chromosome arms - demarcated using a consensus of existing published coordinates [32], [33], [34]); the exceptions are in *S. albus* and *S. griseus.*

**Figure 6:**
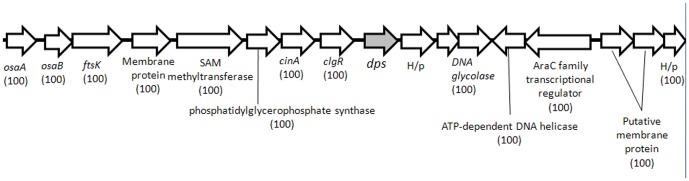
Schematic representation of the genomic neighbourhood of *dpsB_Sc_* in *Streptomyces.* Consensus genomic neighbourhood around *dpsB_Sc_* orthologs (without a *sigB* promoter) in completely sequenced and assembled *Streptomyces* genomes. The nomenclature of genes follows that of *S. coelicolor* orthologs. Numbers in parenthesis represents (as a percentage) the number of times that gene occurs in that position among all the *Streptomyces* tested. Genes that do not match 100% are not shown. H/p  =  hypothetical protein.

The distribution of *dpsB_Sc_* orthologs is more complex. In species where we identified duplicated copies of *dpsB,* at least one of these copies is always located nearer to the ends of the chromosome and this copy always possesses a putative *sigB*-like promoter. The other copy, without the *sigB* promoter is found more centrally in the chromosome. In addition, a comparison of the genomic neighborhood around these paralogs (figure 6) revealed a high degree of gene conservation around the *dps* lacking a putative *sigB*-like promoter. In contrast, very poor gene synteny occurred around the copy where we identified a *sigB*-like promoter motif upstream of corresponding ORFs. Orthologs of *dpsA_Sc_* and *dpsC_Sc_* displayed very poor gene synteny in the genomes analysed.

## Discussion

### The ancestral *dps* in *Streptomyces* possessed short N- and C- terminal tails and was not involved in the osmotic stress response

Our *in silico* predictions, coupled with expression analysis, is suggestive that in many *Streptomyces* there is a requirement for an osmotically inducible *dps.* However, the presence of such a *dps* within the genomes of *Streptomyces* appears fairly recent in evolutionary history. Indeed, we provide evidence that an osmotically inducible *dps* arose either after gene duplication and functional divergence or, in other cases, through the lateral acquisition of an osmotically inducible *dps* from other Actinobacteria. In support of the former, the orthologous relationships in many *Streptomyces* are on a one-to-many basis – e.g. *S. coelicolor* contains a single copy of *dpsB_Sc_*, yet, *S. avermitilis*, *S. scabies*, *S. ghanaensis, S. griseoflavus, S. viridochromogenes* and *S. sviceus* contain two homologous *dpsB* sequences with a high pairwise percentage similarity. The occurrence of two highly similar copies of *dps* in these genomes provides strong evidence for paralogy. Interestingly, a very distinct dichotomy could be observed among paralogous gene pairs based on chromosome location, gene synteny and presence of a putative *sigB* promoter motif. Principally, paralogous pairs could be divided into (i) those that were located within 2 Mb of the chromosome ends, had a poorly conserved genomic neighbourhood and possessed a *sigB*-like promoter motif upstream and (ii), those that were located within the chromosome core, had a highly conserved genomic neighbourhood and did not possess a *sigB*-like promoter motif.

In addition, the presence of a *sigB* promoter motif also conferred expression after osmotic upshock in all paralogs tested. This, coupled with the almost ubiquitous nature of the second class of *dpsB* genes in the genomes of most *Streptomyces* makes them ideal candidates for the ancestral *dps* within this genus. Certainly, in support of this, Bentley’s [32] comparisons between *S. coelicolor*, and *C. diphtheriae* chromosomes indicated that the last common ancestor (LCA) of these taxa may have shared the core region (but not the arms) of the *S. coelicolor* chromosome. Thus, as the core of the *C. diphtheriae* genome, like many Streptomycetes, possesses an ortholog of *dpsB_Sc_*, this is consistent with the hypothesis that the LCA encoded a short- tailed Dps protein and is the ancestral *dps* in the genus *Streptomyces.* Therefore, osmotically-inducible *dpsB_Sc_* orthologs appear to have arisen more recently; possibly as a result of an environmental pressure for osmotic protection. Furthermore, despite the large amount of data implementing Dps tails in the binding of DNA during the stress response, the fact that we show the presence or absence of tails does not correlate with osmotic inducibility suggests that tails are not required for the function of these proteins under osmotic shock.

### Evidence for lateral acquisition of *dps* genes in *Streptomyces*

Phylogenetic reconstruction using all retrieved Dps orthologs indicates that the evolution of Dps proteins in some bacterial species results from HGT rather than by lineal descent. Indeed, HGT has already been demonstrated for *dps* genes elsewhere (e.g. the lactic acid bacterium, *Oenococcus oeni*; [35]) and recent evidence purports that HGT as a mechanism is prevalent in *Streptomyces* [36]. More specifically, it has been shown that the terminal regions at either end of many Streptomycete chromosomes are “hot spots” for laterally acquired genes - with these regions spanning up to 2 Mb in length [32]. Certainly, in *S. coelicolor*, our data are suggestive of HGT as both *dpsA_Sc_* and *dpsC_Sc_* are found within such a region [23]. Together, this would suggest that, at least in *S. coelicolor*, *dpsA* and *dpsC* were additions to the genome. Certainly, orthologs of DpsA_Sc_ are under-represented in other *Streptomyces* species (being found only in *S. coelicolor* and *S. albus*) making the evolution of these orthologs inconsistent with a common ancestry. Interestingly, in agreement with other studies on actinobacterial Dps proteins in the DpsA clade (i.e. that of *Mycobacterium smegmatis*; [37]), DpsA_Sc_ also assembles into two different sized oligomers – suggesting that proteins within this clade may assemble in a similar fashion and could therefore have a similar evolutionary origin. Furthermore, as with *Mycobacterium smegmatis* Dps, the removal of the C-terminal tail of DpsA and N-terminal tail of DpsC in *S. coelicolor* confers an inability of these proteins to assemble correctly (unpublished).

In support of lateral acquisition of *dpsC_Sc_-*like genes, structural and phylogenetic comparisons highlight a clade containing only nine actinobacterial sequences. This is despite the initial BLAST searches using very relaxed E-values. Interestingly, this clade is populated by some species that have been shown to grow in high temperature environments [26]–[29]. Thus, this very narrowly distributed clade contains very interesting Dps proteins. Indeed, *S. coelicolor* DpsC protein dodecamers are highly resistant to denaturation by urea (8 M over night incubation). Our initial modeling studies of this group of proteins (unpublished) suggest that the very long N-terminal tails of these proteins may act to stabilize the dodecamer. As only a small number of species contain orthologs of this gene, they are more likely to have been acquired by horizontal gene transfer rather than having been lost in the majority of species.

The fact that DpsB in *S. coelicolor* does not assemble in our *in vivo* observations is also very interesting. Whilst it is possible that DpsB assembles under different conditions to the other two Dps proteins in *S. coelicolor*, we hypothesise that this protein (and orthologs of this protein in other *Streptomyces*), being the ancestral Dps in this genus, may have become redundant as the number of *dps* genes increased in these genomes. However, further analyses are required to test this.

## Conclusions

In summary, we propose that an osmotically inducible *dps* in *Streptomyces* has resulted from duplications and lateral acquisitions. That *dpsC_Sc_* is very poorly represented in the bacterial lineage is interesting, and this, coupled with the stability of DpsC_Sc_ dodecamers, is suggestive of either very selective acquisition of a gene encoding for a protein with a highly specific function or, significant gene loss. Certainly, the proteins within this clade warrant further study. Moreover, through environmental selection pressure, the genomes of many *Streptomyces* now contain an osmotically induced *dps*. This may have arisen in two ways. Either by duplication, whereby one of the paralogs later becomes part of the osmotically inducible SigB regulon, or, in the absence of such an event, *Streptomyces* have acquired an osmotically induced *dpsA* that is subsequently transcribed as part of the SigB regulon. The order with which these events have occurred is difficult to ascertain. However, the absence of *dpsB* but the presence of *dpsA* with an upstream *sigB*-like promoter motif in *S. flavogriseus* and *S. albus* is suggestive that acquisition of *dpsA* preceded or paralleled duplication of *dpsB* in other species. In those species where an osmotically induced *dps* is absent, or where our *in-silico* analysis has not identified a *sigB* promoter motif upstream of a *dps* ORF (i.e. *S. venezuelae*), this may indicate that there is not a requirement for this trait in the host environment - indeed, *S. venezuelae* is significantly less salt tolerant than *S. coelicolor* (unpublished). Lastly, the variable distribution of *dps* genes in other bacterial genomes is consistent with the patterns we have shown for *Streptomyces*. Thus, we propose that the evolutionary pathway that we describe here, which involves both HGT and gene duplication, may be applicable to the evolution of Dps proteins in many other species.

## Methods

### Bacterial strains, media, RNA isolation and Q RT PCR

*Streptomyces* strains were grown at 30°C on the surface of MS (mannitol soya flour) agar or on cellophane discs [38]. Liquid cultures of nutrient broth were set up in 10 mL volumes. Liquid cultures were incubated at 30°C with shaking (250 r. p. m.). To induce osmotic stress, cellophane disc cultures were grown on MS agar for 16 hours and then transferred to MS agar containing 250 mM KCl. For each species tested, total RNA was isolated from three independent cultures (3 biological replicates), reverse transcription and q RT PCR procedures were performed as previously described [30]. The quantification of *dps* ortholog transcript abundance was performed on five *Streptomyces* species. These were specifically chosen to provide Streptomycetes that, (i) possessed three different Dps proteins (*S. coelicolor*), (ii) duplicated *dps* with and without putative *sigB* promoters (*S. avermitilis* and *S. ghanaensis* and *S. albus*), and (iii) a single *dps*, orthologous to *dpsC_Sc_* (*S. venezuelae*).

Gene specific priming (GSP) of *dps and hrdB/rpoD* was performed using oligonucleotides shown in Table 1. Alignment of the *hrdB* gene sequences showed that the annealing sites of the *hrdB* oligonucleotides used by [19] were conserved among *Streptomyces* spp., thus, these were used to amplify the endogenous reference gene in all samples. Specificity of the reaction was assessed using melt analysis. Fold change in transcript abundance was calculated using the efficiency corrected Pfaffl method [39].

**Table 1:** Plasmids, strains and oligonucleotides.

	Description	Source
*E. coli* BL21 (DE2)	*fhuA2 [lon] ompT gal (λ DE3) [dcm]* Δ*hsdSλ DE3 = λ sBamHIo* Δ*EcoRI-B int::(lacI::PlacUV5::T7 gene1) i21* Δ*nin5*	
E. coli ET12567 (pUZ8002)	Dam13::Tn9 dcm6 hsdM hsdR recF143 16 zjj201::Tn10 galK2 galT22 ara14 lacY1 xyl5 leuB6 thi1 tonA31 rpsL136 hisG4 tsx78 mtli glnV44, containing the non-transmissible oriT mobilizing plasmid, pUZ8002	
*S. coelicolor A3(2)*		
*S. ghanaensis*		
*S. albus*		
*S. venezuelae*		
*S. avermitilis*		
pGEMT-Easy	Ampicillin^R^	Promega corp.
pET26b+	Kanamycin^R^	Novagen
pDpsA4	*dpsA* in pGEM-T Easy	Facey et al., 2009
pDpsC1	*dpsC* in pGEM-T Easy	Facey et al., 2009
pDpsA7 H	*dpsA*::His6, HygromycinR	Facey et a., 2009
pDpsA9	pIJ8600, tipA-*dpsA*::His6	Facey et al., 2009
pDpsB9	pIJ8600, tipA-*dpsB*::His6	Facey et al., 2009
pDpsC9	pIJ8600, tipA-*dpsC*::His6	Facey et al., 2009
pDpA14	*dpsA* coding sequence in pET26b+	This study
pDpsC14	*dpsC* coding sequence in pET26b+	This study
All species	hrdBFor -CCTCCGCCTGGTGGTCTC	Facey et al.,2009
	hrdBRev - CTTGTAGCCCTTGGTGTAGTC	Facey et al., 2009
*S.coelicolor*		
	dpsAF-AGCGGAAGTGGGACGACTAC	Facey *et al.*, 2009
	dpsAR-TCAGAAGGTCCTCGGTGGC	Facey *et al.*, 2009
	dpsBF-GTCGTGAAGAGCCCGTTGTC	Facey *et al.*, 2009
	dpsBR-AGGTGTACGGAGCGGAAGC	Facey *et al.*, 2009
	dpsCF-GGCACCGTCAAGCAGTTCC	Facey *et al.*, 2009
	dpsCR-CCGCCAGGACCTTGTTGAG	Facey *et al.*, 2009
*S. avermitilis*		
	dpsB1F-CTCTCGCTCATCGGGAAG	This study
	dpsB1R-TTCGTCGAGCTGGAGATG	This study
	dpsB2F-GTGGATCTGTCACTCGTG	This study
	dpsB2R-AGTTGCAGGTGAATGGAG	This study
*S. ghanaensis*		
	dpsAF-CATGAAGGAGACCGAGAC	This study
	dpsAR-GACGAACCACTGGAAGAG	This study
	dpsB1F-ATCGACGCCACCGAGAAG	This study
	dpsB1R-GAACATCCACCGCTGCTT	This study
	dpsB2F-TCGTGAAGAGTCCATTGTC	This study
	dpsB2R-CGAGGGCGAGATCCACCAG	This study
*S. venezuelae*		
	dpsCF-CTCAACCTGCCGAACAAG	This study
	dpsCR-AAGTACACCTCGTTGTCGTT	This study
*S. albus*		
	dpsAF-AAGCTCATCGACCTCCTG	This study
	dpsAR-CCAGTGGATGTGCTTCAG	This study
	dpsBF-AGCCAGGACATCTTCATCA	This study
	dpsBR-CTAGCTGTTCTCCGCCTG	This study

### Obtaining an othologous data set of Dps proteins

An actinobacterial orthologous data set of Dps*_Sc_* proteins was obtained using a Reciprocal Best-Hit (RBH; [40]) approach. Briefly, the three *S. coelicolor* Dps protein sequences were used as queries for retrieving homologous sequences using the Blastp algorithm [41] and a relaxed E value threshold of 1E^−03^. Default parameters were used throughout - however, to limit data redundancy, we retrieved sequences from the refseq database. Results from these initial searches were then re-blasted back limiting results to *Streptomyces coelicolor.* Only proteins that came out as best hits bi-directionally were retained. Sequences of retrieved proteins were aligned using ClustalW implemented in Molecular Evolutionary Genetics Analysis software (MEGA; [42]). Moreover, to correct for erroneous annotations and to exclude bacterioferritins from the orthologous data list, firstly, a distance based (Neighbor-joining) tree was reconstructed that included all putative Dps orthologs and *Streptomyces coelicolor* bacterioferritin (Genbank: NP626370) and secondly, the secondary structure of all proteins were predicted using JPRED3 [43]. All secondary structure annotations were scrutinized and poorly predicted structures (i.e. those with confidence levels lower than 40%) were manually removed. Sequences were excluded from the data set if they grouped together with *S. coelicolor* bacterioferritin and also lacked the characteristic BC helix of Dps (i.e. those sequences that were bacterioferritins/ not Dps). In addition, to characterize Dps homologs in terms of N- and C- terminal tail length, using the secondary structure predictions, we manually counted the number of amino acid upstream of the first and downstream of the last alpha helix for each protein.

### Gene synteny and chromosome location of *dps* genes in completely sequenced *Streptomyces* genomes

In the absence of horizontal gene transfer and duplication, the chromosome location of orthologous genes and gene synteny surrounding them is often conserved amongst closely related species. Thus, we chose to compare location of *dps* genes in 17 completely sequenced and assembled *Streptomyces* genomes. In addition, we manually compared gene synteny of genes surrounding *dps.* Chromosome coordinates of orthologous *dps* genes used to construct gene location maps were retrieved from NCBI. To calibrate chromosome orientation, we chose to use the direction of the initiator protein, *dnaA,* located in the central *oriC* region of streptomycete chromosomes [44], [45].

### Phylogenetic reconstruction

A Maximum-likelihood based phylogeny using Actinobacterial Dps ortholog sequences was reconstructed using MEGA using the amino acid substitution model JTT + F +I. The consensus tree was drawn using the Majority Rule criteria. To assess the robustness of inferred phylogenies, we used 500 bootstrap pseudoreplicates.

### Identification of SigB dependent promoters upstream of *dps* genes

The conservation of a *sigB*-like regulon among *dps* orthologs in Streptomycete*s* was investigated using an *in-silico* approach. An *in silico* search using the degenerative motif GNNTN(N) 14–16 GGGYAY was performed on the complete genomic sequences of 17 Streptomycetes using COMplex PAttern of sequence search software (COMPAsss; [46]). Organisms were only included in the analysis if they were classified down to the species level. The occurrence of this motif, which represents the −10 and −35 regions of the *dpsA*/*sigB* recognizable promoter [30] in the 17 *Streptomyces* genomes, was retrieved using the COMPAsss built-in database mining capability linked to the NCBI nucleotide database. Generated hit tables were scrutinized for putative functional promoters by limiting hits to those found in intergenic regions and to motifs located within 200 nucleotides of *dps* ORFs.

### Protein methods

Assembly of *S. coelicolor* Dps into higher state oligomers was investigated *in vivo* using C-terminally His-tagged *dpsA_Sc_*, *dpsB_Sc_* and *dps*C*_Sc_* under the control of a thiostrepton inducible promoter to ensure sufficient protein concentration levels, encoded respectively by plasmids pDpsA9, pDpsB9 and pDpsC9 [19]. For overexpression, 24-hour liquid cultures were spiked with the antibiotic thiostrepton (25 µg ml^−1^) for protein induction and incubated for a further hour. Cells were pelleted by centrifugation (5 000 x g for 5 min) and the media replaced with sonication buffer (50 mM Tris-HCl, pH 8, 200 mM NaCl, 15 mM EDTA, Complete protease inhibitor cocktail (Roche Diagnostics). Cells were disrupted by sonication on ice (20 seconds at 20% amplitude). Cell free extracts were obtained by centrifugation (13 000 x g for 2 min at 4°C) and removal of the supernatant. Equal volumes of supernatant were mixed with NativePAGE sample buffer (Invitrogen) and proteins were separated in a 7% polyacrylamide gel using a tris-glycine running buffer excluding sodium dodecyl sulphate (SDS) at 4°C. After electrophoresis, gels were soaked in 1 X SDS running buffer for 30 min and then equilibrated for 5 min in cold Bjerrum and Schafer-Nielsen transfer buffer. Proteins were transferred to a polyvinylidene difluoride (PVDF) membrane (Hybond-P, Amersham) using a semi-dry electrophoretic transfer cell (Trans-Blot SD, BioRad). His-tagged proteins were detected with a Penta- His peroxidase conjugate (Qiagen). Immunological detection was performed using an ECL Advance Western blotting detection kit (Amersham Pharmacia Biotech). The stability of assembled *S. coelicolor* Dps dodecamers was investigated using recombinantly expressed Dps. Briefly, C-terminal translational fusions to 6xhistidine tag were created as follows: the coding sequences for *dpsA_Sc_* and *dpsC_Sc_* were excised from plasmids pDpsA4 and pDpsC1 [19] as NdeI/BglII fragments and subcloned into pET26b(+) digested NdeI/BglII to create pDpA14 and pDpsC14 respectively. Plasmids were transformed into *E. coli* BL21 (DE3) and grown in 1 L cultures of 2 X YT until mid-log phase. Expression of recombinant Dps proteins was performed at 30°C for 3 h after the addition of isopropyl-1-thio-D-galactoside (IPTG) to a final concentration of 0.1 mM. Cells were harvested by centrifugation and resuspended in a sonication buffer (20 mM Tris/HCl, 500 mM NaCl, 50 mM Imidazole, Complete protease inhibitor cocktail [Roche Diagnostics], pH 7.5) and disrupted by sonication. A cell-free supernatant was applied to a Ni Sepharose High Performance column (HisTrap HP; GE Healthcare). Fractions containing Dps proteins were pooled and buffer exchanged using HiTrap (GE Healthcare) into 20 mM, 200 mM NaCl and 5% Glycerol. To assess stability, purified proteins were mixed with Urea (8 M) and incubated overnight; followed by native PAGE. Proteins were visualised using Coomassie Blue staining.

## References

[1] Matin A (1991) The molecular basis of carbon-starvation-induced general resistance in Escherichia coli. Molecular Microbiology 5: 3–10. 2014002 10.1111/j.1365-2958.1991.tb01819.x

[2] Almirón M, Link AJ, Furlong D, Kolter R (1992) A novel DNA-binding protein with regulatory and protective roles in starved Escherichia coli. Genes & Development 6: 2646–2654. 1340475 10.1101/gad.6.12b.2646

[3] Chowdhury RP, Saraswathi R, Chatterji D (2010) Mycobacterial stress regulation: The Dps "twin sister" defense mechanism and structure-function relationship. IUBMB Life 62: 67–77. 20014234 10.1002/iub.285

[4] Pesek J, Büchler R, Albrecht R, Boland W, Zeth K (2011) Structure and Mechanism of Iron Translocation by a Dps Protein from Microbacterium arborescens. Journal of Biological Chemistry 286: 34872–34882. 21768097 10.1074/jbc.M111.246108 PMC3186433

[5] Calhoun LN, Kwon YM (2010) Structure, function and regulation of the DNA-binding protein Dps and its role in acid and oxidative stress resistance in Escherichia coli: a review. J Appl Microbiol 110: 375–386. 21143355 10.1111/j.1365-2672.2010.04890.x

[6] Chiancone E, Ceci P (2010) The multifaceted capacity of Dps proteins to combat bacterial stress conditions: Detoxification of iron and hydrogen peroxide and DNA binding. Biochim Biophys Acta 1800: 798–805. 20138126 10.1016/j.bbagen.2010.01.013

[7] Peña MMO, Bullerjahn GS (1995) The DpsA Protein of Synechococcus sp. Strain PCC7942 Is a DNA-binding Hemoprotein. Journal of Biological Chemistry 270: 22478–22482. 7673237 10.1074/jbc.270.38.22478

[8] Takatsuka M, Osada-Oka M, Satoh EF, Kitadokoro K, Nishiuchi Y, et al (2011) A Histone-Like Protein of Mycobacteria Possesses Ferritin Superfamily Protein-Like Activity and Protects against DNA Damage by Fenton Reaction. PLoS One 6: e20985. 21698192 10.1371/journal.pone.0020985 PMC3116847

[9] Grant RA, Filman DJ, Finkel SE, Kolter R, Hogle JM (1998) The crystal structure of Dps, a ferritin homolog that binds and protects DNA. Nat Struct Biol 5: 294–303. 9546221 10.1038/nsb0498-294

[10] Kim SG, Bhattacharyya G, Grove A, Lee YH (2006) Crystal structure of Dps-1, a functionally distinct Dps protein from Deinococcus radiodurans. J Mol Biol 361: 105–114. 16828801 10.1016/j.jmb.2006.06.010

[11] Andrews SC (2010) The ferritin-like superfamily: Evolution of the biological iron storeman from a rubrerythrin-like ancestor. Biochima et Biophysica Acta: 691-705. 10.1016/j.bbagen.2010.05.010 20553812

[12] Zhang Y, Orner BP (2011) Self-Assembly in the Ferritin Nano-Cage Protein Superfamily. International Journal of Molecular Sciences 12: 5406–5421. 21954367 10.3390/ijms12085406 PMC3179174

[13] Fan R, Boyle AL, Cheong W, Ng Sl, Orner BP (2009) A helix swapping study of two protein cages. Biochemistry 48: 5623–5630. 19405543 10.1021/bi900387t

[14] Haikarainen T, Papageorgiou AC (2010) Dps-like proteins: structural and functional insights into a versatile protein family. Cell Mol Life Sci 67: 341–351. 19826764 10.1007/s00018-009-0168-2 PMC11115558

[15] Bhattacharyya G, Grove A (2007) The N-terminal extensions of Deinococcus radiodurans Dps-1 mediate DNA major groove interactions as well as assembly of the dodecamer. J Biol Chem 282: 11921–11930. 17331944 10.1074/jbc.M611255200

[16] Roy S, Saraswathi R, Gupta S, Sekar K, Chatterji D, et al (2007) Role of N and C-terminal tails in DNA binding and assembly in Dps: structural studies of Mycobacterium smegmatis Dps deletion mutants. J Mol Biol 370: 752–767. 17543333 10.1016/j.jmb.2007.05.004

[17] Su M, Cavallo S, Stefanini S, Chiancone E, Chasteen ND (2005) The so-called Listeria innocua ferritin is a Dps protein. Iron incorporation, detoxification, and DNA protection properties. Biochemistry 44: 5572–5578. 15823015 10.1021/bi0472705

[18] Ishikawa T, Mizunoe Y, Kawabata S, Takade A, Harada M, et al (2003) The iron-binding protein Dps confers hydrogen peroxide stress resistance to Campylobacter jejuni. J Bacteriol 185: 1010–1017. 12533477 10.1128/JB.185.3.1010-1017.2003 PMC142835

[19] Facey PD, Hitchings MD, Saavedra-Garcia P, Fernandez-Martinez L, Dyson PJ, et al (2009) Streptomyces coelicolor Dps-like proteins: differential dual roles in response to stress during vegetative growth and in nucleoid condensation during reproductive cell division. Mol Microbiol 73: 1186–1202. 19719512 10.1111/j.1365-2958.2009.06848.x

[20] Nair S, Finkel SE (2004) Dps protects cells against multiple stresses during stationary phase. J Bacteriol 186: 4192–4198. 15205421 10.1128/JB.186.13.4192-4198.2004 PMC421617

[21] Thieme D, Grass G (2010) The Dps protein of Escherichia coli is involved in copper homeostasis. Microbiol Res 165: 108–115. 19231146 10.1016/j.micres.2008.12.003

[22] Choi SH, Baumler DJ, Kaspar CW (2000) Contribution of dps to acid stress tolerance and oxidative stress tolerance in Escherichia coli O157:H7. Appl Environ Microbiol 66: 3911–3916. 10966408 10.1128/aem.66.9.3911-3916.2000 PMC92238

[23] Nicodeme M, Perrin C, Hols P, Bracquart P, Gaillard JL (2004) Identification of an iron-binding protein of the Dps family expressed by Streptococcus thermophilus. Curr Microbiol 48: 51–56. 15018103 10.1007/s00284-003-4116-3

[24] Roy S, Saraswathi R, Chatterji D, Vijayan M (2008) Structural studies on the second Mycobacterium smegmatis Dps: invariant and variable features of structure, assembly and function. J Mol Biol 375: 948–959. 18061613 10.1016/j.jmb.2007.10.023

[25] Schwartz JK, Liu XS, Tosha T, Diebold A, Theil EC, et al. CD and MCD spectroscopic studies of the two Dps miniferritin proteins from Bacillus anthracis: role of O2 and H2O2 substrates in reactivity of the diiron catalytic centers. Biochemistry 49: 10516–10525. 10.1021/bi101346c PMC3075618 21028901

[26] Reon BJ, Nguyen KH, Bhattacharyya G, Grove A (2012) Functional comparison of Deinococcus radiodurans Dps proteins suggests distinct in vivo roles. Biochemical Journal Epub ahead of print. 10.1042/BJ20120902 22857940

[27] Saraswathi R, Pait Chowdhury R, Williams SM, Ghatak P, Chatterji D (2009) The Mycobacterial MsDps2 Protein Is a Nucleoid-Forming DNA Binding Protein Regulated by Sigma Factors σA and σB. PLoS One 4: e8017. 19956571 10.1371/journal.pone.0008017 PMC2779847

[28] Gupta S, Pandit SB, Srinivasan N, Chatterji D (2002) Proteomics analysis of carbon-starved Mycobacterium smegmatis: induction of Dps-like protein. Protein Eng 15: 503–512. 12082169 10.1093/protein/15.6.503

[29] Roy S, Gupta S, Das S, Sekar K, Chatterji D, et al (2003) Crystallization and preliminary X-ray diffraction analysis of Mycobacterium smegmatis Dps. Acta Crystallogr D Biol Crystallogr 59: 2254–2256. 14646086 10.1107/s0907444903018742

[30] Facey PD, Sevcikova B, Novakova R, Hitchings MD, Crack JC, et al (2011) The dpsA Gene of Streptomyces coelicolor: Induction of Expression from a Single Promoter in Response to Environmental Stress or during Development. PLoS One 6: e25593. 21984935 10.1371/journal.pone.0025593 PMC3184153

[31] Chowdhury RP, Gupta S, Chatterji D (2007) Identification and characterization of the dps promoter of Mycobacterium smegmatis: promoter recognition by stress-specific extracytoplasmic function sigma factors sigmaH and sigmaF. J Bacteriol 189: 8973–8981. 17921287 10.1128/JB.01222-07 PMC2168604

[32] Bentley SD, Chater KF, Cerdeno-Tarraga AM, Challis GL, Thomson NR, et al (2002) Complete genome sequence of the model actinomycete Streptomyces coelicolor A3(2). Nature 417: 141–147. 12000953 10.1038/417141a

[33] Fisher G, Decaris B, Leblond P (1997) Occurrence of deletions, associated with genetic instability in Streptomyces ambofaciens, is independent of the linearity of the chromosomal DNA. Journal of Bacteriology 179: 4553–4558. 9226265 10.1128/jb.179.14.4553-4558.1997 PMC179291

[34] Choulet F, Aigle B, Gallois A, Mangenot S, Gerbaud C, et al (2006) Evolution of the Terminal Regions of the Streptomyces Linear Chromosome. Molecular Biology and Evolution 23: 2361–2369. 16956972 10.1093/molbev/msl108

[35] Athane A, Bilhere E, Bon E, Morel G, Lucas P, et al (2008) Characterization of an acquired dps-containing gene island in the lactic acid bacterium Oenococcus oeni. J Appl Microbiol 105: 1866–1875. 19120635 10.1111/j.1365-2672.2008.03967.x

[36] Doroghazi JR, Buckley DH (2010) Widespread homologous recombination within and between Streptomyces species. ISME J 4: 1136–1143. 20393569 10.1038/ismej.2010.45

[37] Gupta S, Chatterji D (2003) Bimodal Protection of DNA by Mycobacterium smegmatis DNA-binding Protein from Stationary Phase Cells. Journal of Biological Chemistry 278: 5235–5241. 12466274 10.1074/jbc.M208825200

[38] Kieser T, Bibb MJ, Buttner MJ, Chater KF, Hopwood DA (2000) Practical Streptomyces Genetics. Norwich: John Innes Foundation.

[39] Pfaffl MW (2001) A new mathematical model for relative quantification in real-time RT–PCR. Nucleic Acids Research 29: e45–e45. 11328886 10.1093/nar/29.9.e45 PMC55695

[40] Moreno-Hagelsieb G, Latimer K (2008) Choosing BLAST options for better detection of orthologs as reciprocal best hits. Bioinformatics 24: 319–324. 18042555 10.1093/bioinformatics/btm585

[41] Altschul SF, Gish W, Miller W, Myers EW, Lipman DJ (1990) Basic local alignment search tool. Journal of Molecular Biology 215: 403–410. 2231712 10.1016/S0022-2836(05)80360-2

[42] Tamura K, Peterson D, Peterson N, Stecher G, Nei M, et al.. (2011) MEGA5: Molecular Evolutionary Genetics Analysis using Maximum Likelihood, Evolutionary Distance, and Maximum Parsimony Methods. Molecular Biology and Evolution. 10.1093/molbev/msr121 PMC3203626 21546353

[43] Cole C, Barber JD, Barton GJ (2008) The Jpred 3 secondary structure prediction server. Nucleic Acids Research 36: W197–W201. 18463136 10.1093/nar/gkn238 PMC2447793

[44] Smulczyk-Krawczyszyn A, Jakimowicz D, Ruban-Ośmiałowska B, Zawilak-Pawlik A, Majka J, et al (2006) Cluster of DnaA Boxes Involved in Regulation of Streptomyces Chromosome Replication: from In Silico to In Vivo Studies. Journal of Bacteriology 188: 6184–6194. 16923885 10.1128/JB.00528-06 PMC1595370

[45] Hopwood DA (2006) Soil To Genomics: The Streptomyces Chromosome. Annu Rev Genet 40: 1–23. 16761950 10.1146/annurev.genet.40.110405.090639

[46] Maccari G, Gemignani F, S L (2010) COMPASSS (COMplex PAttern of Sequence Search Software), a simple and effective tool for mining complex motifs in whole genomes. Bioinformatics 26: 1777–1778. 20501554 10.1093/bioinformatics/btq258

